# IMAGINING THE FUTURE OF AGETECH

**DOI:** 10.1093/geroni/igad104.1600

**Published:** 2023-12-21

**Authors:** Andrew Sixsmith

## Abstract

The intersection of global population aging and technological trends in e-health, robotics, artificial intelligence, and mobile technologies has led to the emergence of “AgeTech” as a way to improve the health and well-being of older people. While this might be seen as an almost deterministic process of technological change, the visioning, development and implementation of technology is essentially a creative and socially-situated process. This requires us to understand AgeTech as a socio-technical imaginary, examining the intersecting spheres of social action and technological development. This idea of technological development as a creative process, runs counter to an idea of technology in purely rational and instrumental terms and brings into play cultural, political and behavioural factors. Our symposium examines the socio-technical imaginary of AgeTech in three papers: Paul Higgs and Chris Gilleard discuss Fourth Ageism and the Technological Imaginary of Long-term care and argue that the problematizing of aging present in current thinking needs to be challenged at the conceptual stage of technological development. Andrew Sixsmith et al will present on A Global AgeTech Agenda, with a particular focus on how AgeTech development and innovation should connect with global initiatives such as the UN’s Strategic Development Goals. Mei Lan Fang et al will take an Ethical Perspective on AgeTech to discuss some of the complex challenges around the creation and use of technologies and argue that culture change is needed within the research and innovation communities to develop ethically grounded AgeTech. This is a Technology and Aging Interest Group Sponsored Symposium.

